# Procalcitonin levels in preterm newborns: Reference ranges during the first three days of life

**DOI:** 10.3389/fped.2022.925788

**Published:** 2022-08-29

**Authors:** Cristina Tuoni, Massimiliano Ciantelli, Riccardo Morganti, Martina Violi, Sara Tamagnini, Luca Filippi

**Affiliations:** ^1^Neonatology and Neonatal Intensive Care Unit, Department of Clinical and Experimental Medicine, University Hospital of Pisa, Pisa, Italy; ^2^SOD Clinical Trial Statistical Support, Azienda Ospedaliero Universitaria Pisana, Pisa, Italy

**Keywords:** procalcitonin (PCT), early onset sepsis (EOS), very low birth weight (VLBW), preterm/full term infants, reference range intervals

## Abstract

**Background:**

Sepsis is one of the most important causes of morbidity and mortality in the neonatal period, especially in preterms. Diagnosis is difficult because of specific signs and symptoms. The diagnostic gold standard is blood culture, but its sensibility is low. Much effort has been made to identify early, sensitive, and specific diagnostic markers; among these markers particular attention was paid to procalcitonin. However, reference ranges of serum procalcitonin (PCT) shortly after birth have not been sufficiently studied in healthy preterms, and literature is still contradictory.

**Objectives:**

The aim of the study is to define PCT age-specific reference ranges in the first 72 h of life in uninfected VLBW preterms.

**Methods:**

Serum levels of PCT were assessed for each newborn at birth and every 24 h until the 3rd day of life. The eligible patients were classified into two groups according to their sepsis status.

**Results:**

Approximately 343 patients were enrolled; 28 were septic and 315 non-septic. In non-septic infants, 1,015 determinations of PCT values were performed. Our data showed a trend in average value of PCT to increase after birth up to a peak between 24 and 48 h of life and, subsequently, to fall. The average peak value was 15.12 ng/ml achieved at nearly 36 h of life.

**Conclusion:**

Our study shows a PCT nomogram of healthy preterms, which is different from the one of term newborns. Data agree with what is reported in literature on the reference ranges and trends of PCT in non-septic preterms shortly after birth.

## Introduction

Neonatal sepsis is a clinical syndrome characterized by systemic signs and symptoms of infection and by the presence of pathogen agents in blood, which occurs in the first 28 days of life (1).

Neonatal sepsis still represents an important cause of mortality and morbidity among infants, above all in very low birth weight (VLBW, birth weight < 1,500 g) preterm infants, with an incidence ranging from 1 to 20/1,000 live births (2), with a mortality of 11–19% and unquantified long-term neurological defects (3).

According to time of infection, we can distinguish early onset sepsis (EOS), during the first 72 h of life, and late onset sepsis (LOS) after 72 h from delivery.

Neonatal sepsis presents common and unspecific clinical manifestations so that an infant with a suspect for sepsis requires rapid assessment to guarantee rapid therapy. Blood culture remains the gold standard for diagnosis, but data available from two large randomized controlled trials in recent years have shown culture-negative sepsis rates of 56 and 46%, respectively (4).

Blood culture negative, clinically diagnosed sepsis accounts for the majority of these cases, which is problematic because the definition of clinical sepsis is variable and often includes subjective signs on the physical exam (5).

There are many different biomarkers that have been discovered to be of help in the early diagnosis of neonatal sepsis. Nevertheless, high variability in sensitivity and specificity of those markers is reported in literature; this may be due to many factors, such as gestational age, birth weight, cut-off used, and time of blood sampling. Presently, procalcitonin (PCT) and CRP are the most commonly used and have been studied in numerous neonates (6).

Procalcitonin usefulness in EOS remains uncertain, despite its wide use in LOS. PCT differs from CRP for a faster kinetic: Its release from tissues increases during infections more rapidly than CRP and can be detected in circulation within 2–4 h, with peak concentrations achieved after 6–8 h and a half-life of 20–24 h (7).

Several studies have reported the usefulness of serum procalcitonin (PCT) as a potential biomarker for bacterial infections in adults and children, and the reference range is less than 0.1 ng/ml, while the cut-off value for the diagnosis of bacterial infections or sepsis has been established at 0.5 ng/ml. During bacterial or fungal infections, the PCT increased up to 2 ng/ml or even 50–100 ng/ml (8).

These values concern children but not newborns during the 1st days of life, when we can find a physiologic peak of PCT in non-infected newborns (which, in many cases, is more evident 24 h after birth) (9), which makes difficult the interpretation of laboratory data in the first 72 h of life.

The PCT reference range during the neonatal period varies greatly depending on gestational age and infectious status. Its values in EOS are considerably higher than those of non-infected infants, and many studies report different age-related nomograms for both term infants and preterms (9–12). Anyway, the literature highlights a huge heterogeneity between studies and low methodological quality due to: arbitrary cut-offs used to differentiate infectious and non-infectious conditions; heterogeneity in study groups; PCT response assessed in neonates with different postnatal ages without consideration of gestational age (GA) and birth weight (BW); absence of data on how severity of underlying illness might have hampered PCT response. Therefore, more studies are needed to determine its usefulness in diagnosing EOS.

In our study, we investigated the 95% age-specific reference intervals of PCT values in the first 72 h of life in VLBW preterm infants.

## Patients and methods

Our study population consists of 343 preterm newborns enrolled over 4 years (2017–2020) in the Neonatal Intensive Care Unit (NICU) of Santa Chiara University Hospital of Pisa (Italy). Inclusion criteria were GA ≤ 32 weeks + 6 days and/or birth weight ≤ 1,500 g. Absolute exclusion criteria were exitus within 48 h of life, asphyxia, outbirth with transfer to our Center after 4 h of life and absence of informed consent.

The patients were divided into 2 groups based on the presence or absence of EOS: (1) non-septic and (2) septic. In Group 2 were included infants with:

– Certain sepsis: positive blood culture performed at birth and clinical/radiographic signs of infection.
– Clinical sepsis: negative blood culture performed at birth but clinical signs of sepsis, obstetric risk factors of infection, positive skin swab culture performed at birth or a positive bronchial aspirate culture if intubated, positive sepsis screening (presence of at least 2 between leukocytosis/leukopenia, neutrophilia, and CRP ≥ 1 mg/dl) and/or radiological signs of pneumonia.

To all enrolled patients, empirical antibiotic therapy (ampicillin + sulbactam and gentamicin) was administered from birth to 72 h if EOS was excluded or prolonged for 7–14 days in relation to sepsis.

Procalcitonin measurements were performed at birth and every 24 ± 4 h until the 3rd day of life. For all patients were recorded gender, GA, BW, weight percentile, 5-min Apgar score, intubation in delivery room. In combination with PCT, it was always measured CRP. In all newborns, we also examined WBC, liver and kidney function, blood and skin swabs culture, and chest X-ray at birth.

### Procalcitonin determinations

Procalcitonin serum assays were performed on samples of blood taken with microtubes in K2-EDTA or in lithium-heparin. The PCT blood concentration was measured by the immunometric electrochemiluminescence technique (Elecsys BRAHMS PCT instrument). According to the EP05-A protocol of the Clinical and Laboratory Standards Institute (CLSI), the inter- and intra-assay coefficients of variation (CV) were, respectively, ≤2.1 and 2.4%.

### Statistical analysis

In accordance with the literature, we analyzed the natural logarithm of PCT values (lnPCT). To determine the PCT reference range in the 1st 72 h of life in non-septic preterm infants, we built a graph with the 95% CI. To compare means of two independent groups, we used *t*-test for independent samples (two-tailed).

Significance was defined as *p* < 0.05. SPSSv.27 technologies were applied.

## Results

Over the study period, we enrolled 364 preterm infants. Among them, 6 died within the 1st 24 h of life and were not included in the study. Of the 358 remaining patients, 15 were excluded due to perinatal asphyxia. The analysis was drawn up on 343 infants, of which 163 were girls and 180 were boys. Of these, 315 were non-septic, and 28 were septic. The two groups resulted statistically homogeneous relatively to GA, BW, and gender.

The comparison of lnPCT values at 24 ± 4 h of life (t1) showed statistically significant differences between the 2 groups (PCTt1, 97.66 vs. 15.12; *t* = –5.138, *p* < 0.001), with higher values in septic patients. Similar results were obtained for lnPCT values at birth (t0) (PCTt0, 7.21 vs.36; *t* = –2.48; *p* < 0.001), at 48 ± 4 h of life (t2) (PCTt2, 82.56 vs. 6.68; *t* = –6.382; *p* < 0.001) and at 72 ± 4 h of life (t3) (PCTt3, 27.42 vs. 2.16; *t* = –2.665; *p* < 0.001).

### Construction of the reference range

**FIGURE 1:**
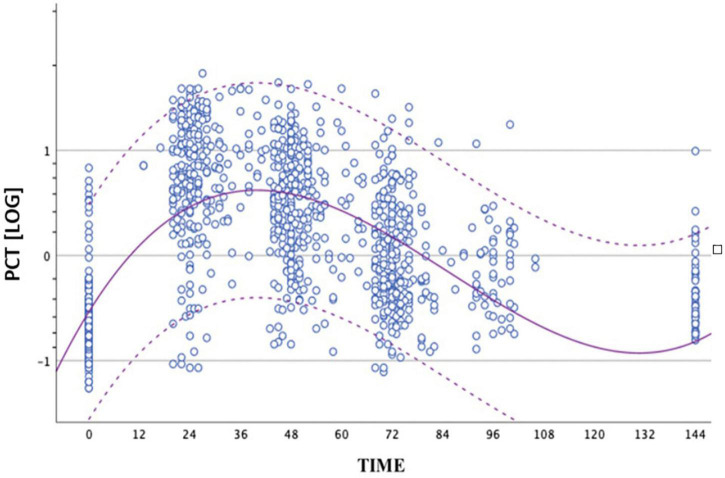
Distribution of PCT values obtained from the group of non-septic newborns between birth and 72 ± 4 h of life. The circles represent the logPCT values. The dashed curves represent the upper and lower limits of the reference range. The continuous curve represents the geometric mean. The logarithmic scale of the PCT values is shown on the ordinate axis.

We performed 1,015 determinations of PCT values in non-septic infants group in the first 72 h of life. We built the distribution curve of PCT using these values. Our data showed a trend in average value of PCT to increase after birth up to a peak between 24 and 48 h of life and, subsequently, to fall (Figure 1). We constructed graphs using a cubic model. The average peak value was 15.12 ng/ml achieved at nearly 36 h of life.

**FIGURE 2:**
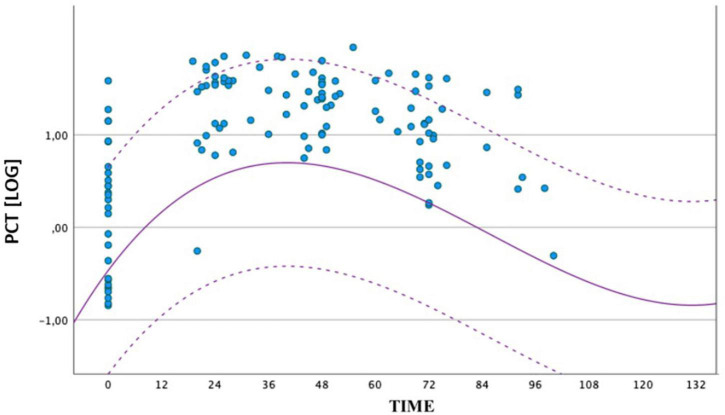
Comparison between the distribution of PCT values obtained by the group of septic newborns between birth and 72 ± 4 h of life. The circles represent the individual logPCT values of septic infants, while the dashed curves represent the reference range of non-septic infants. The logarithmic scale of the PCT values is shown on the ordinate axis.

Because of statistically significant difference of PCT values at all times between the two groups, we also compared the distribution of PCT values obtained in the septic group (circles) and the geometric mean, with upper and lower limits of the reference range of the non-septic group (dashed curves). All the values of the septic group at peak were higher than the mean of the non-septic group (Figure 2).

**FIGURE 3:**
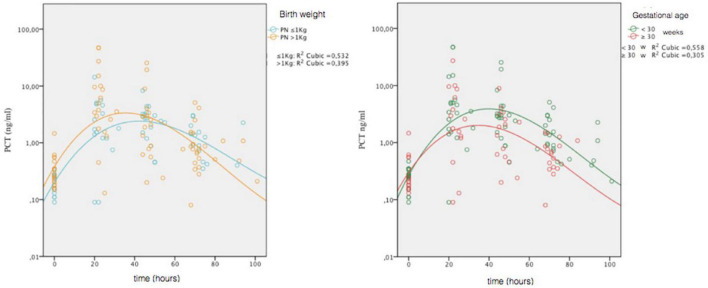
Comparison of PCT values obtained in the four non-septic group (gestational age, ≥30 weeks and <30 weeks; birth weight, >1,000 and ≤1,000 g). Circles represent individual values; combined lines represent geometric mean. On the ordinate is the natural logarithmic scale of PCT values.

To assess any differences in the PCT trend, depending on GA and BW, we stratified the non-septic group on two categories of GA (≥30 weeks and <30 weeks) and two categories of BW (>1,000 g and ≤1,000 g), and we constructed reference curves for all groups. We also examined whether there were significant differences for the presence of SGA in the four categories. It was found an increased number of SGA in the BW > 1,000-g category at limits of statistical significance (*p* = 0.052). For the GA ≥ 30-week group, the peak was 4.81 ng/ml at 29 h. For the GA < 30 weeks group, the peak was 8.86 ng/ml at 32 h. For the BW > 1,000-g group, the peak was 10.34 ng/ml at 31 h. For the BW ≤ 1,000-g group, the peak was 3.20 ng/ml at 36 h. In Figure 3, we compared the curves of four groups.

We made comparison of lnPCT values at t1 and t2 for categories of GA and BW. The only significant difference was detected between GA categories at t2 (*t* = 2.270; *p* = 0.030), with higher values in the GA < 30 weeks group.

## Discussion

The main purpose of this study was to determine age-related reference intervals for PCT in VLBW preterms during the early neonatal period because there is, still, a huge heterogeneity between studies in literature.

Chiesa et al. reported a peak of 20 ng/ml for healthy term infants at 24 h of life; (10) Altunhan et al. did not distinguish between term and preterm newborns and described a peak of 5.38 ng/ml in the first day of life in septic patients; (11) more recently, a Turkish study has reported in the 1st few hours of life a cut-off value of 1.4 ng/ml for preterm septic newborns with a gestational age between 23 and 36 week (11), and a Japanese study described a cut-off value at 24 h of life of 11.1, 1.2, and 2.2 ng/ml, respectively, for VLBW, late-preterm, and term babies (12).

In our study, a statistically significant difference in PCT values between septic and non-septic patients was found out at all times analyzed. This difference let us construct an age-related nomogram for PCT concentration in a large cohort of non-infected VLBW preterm infants.

Regarding PCT values and the trend in non-septic infants in the 1st 3 days of life, our data agree with those reported in literature with a clear tendency to increase up to a peak after birth and then gradually decrease. The average peak value was 15.12 ng/ml achieved at nearly 36 h of life, a value that tends to be higher than those reported in literature.

Other studies show differences in PCT concentration according to gestational age (12), so we decided to stratify the nomograms according to gestational age and birth weight too. Reference curves stratified by weight and age show that at lower gestation, ages correspond highest, later and longer peaks, and at lower weights, peaks are lower and later. Despite GA and BW are variables closely related, the trend of PCT is different between smaller GA and BW categories. Partly, this is possibly due to greater presence of SGA infants in our BW > 1,000-g group compared to that with weight ≤1,000 g, even if it is only borderline significance (*p* = 0.052).

Reports on preterm infants suggested that the physiological rise in PCT concentration of preterm infants may be longer and higher than in term infants. In our cohort, the peak value was actually longer but not higher than that reported in literature for term infants (9, 10), and values at 72 h of life were higher, taking longer to return to normal values. These results could be explained in immature systems and metabolism of the preterm newborns, resulting in delayed kinetics.

## Conclusion

In conclusion, we reported reference ranges for serum PCT concentrations in healthy VLBW preterm newborns during the first 3 days after birth, and we developed an age-related nomogram.

A major limitation of this study is that we did not investigate how the severity of underlying illnesses might have hampered PCT response nor the possible influence of newborns factors, such as respiratory failure, which, in literature, is reported as the main effect or for the transient elevation in serum PCT levels in 1st days of life (13). So further studies are needed to confirm the accuracy of this nomogram.

## Data availability statement

The raw data supporting the conclusions of this article will be made available by the authors, without undue reservation.

## Ethics statement

The studies involving human participants were reviewed and approved by the Regional Ethics Committee of Tuscany – Pediatric Section. Written informed consent to participate in this study was provided by the participants’ legal guardian/next of kin.

## Author contributions

MC conceptualized and designed the study. CT drafted the initial manuscript and reviewed and revised the manuscript. MV and ST collected the data. RM carried out the analyses. LF reviewed and revised the manuscript. All authors approved the final manuscript as submitted and agree to be accountable for all aspects of the work.

## References

[1] Gomella TL. *Neonatology: Management, Procedures, On-Call Problems, Diseases, and Drugs - Seventh Edition.* New York, NY: McGraw-Hill Education/Medical (2013).

[2] Odabasi IO Bulbul A. Neonatal Sepsis. *Med Bull Sisli Etfal Hosp.* (2020) 54:142–58. 10.14744/SEMB.2020.00236 PMC7326682 32617051

[3] Molloy EJ Wynn JL Bliss J Koenig JM Keij FM McGovern M Neonatal sepsis: need for consensus definition, collaboration and core outcomes. *Pediatr Res.* (2020) 88:2–4. 10.1038/s41390-020-0850-5 32193517

[4] Hayes R Hartnett J Semova G Murray C Murphy K Carroll L Infection, inflammation, immunology and immunisation (I4) section of the european society for paediatric research (ESPR). Neonatal sepsis definitions from randomised clinical trials. *Pediatr Res.* (2021). 10.1038/s41390-021-01749-3 [Epub ahead of print].

[5] Eschborn S Weitkamp JH. Procalcitonin versus C-reactive protein: review of kinetics and performance for diagnosis of neonatal sepsis. *J Perinatol.* (2019) 39:893–903. 10.1038/s41372-019-0363-4 30926891

[6] Sharma D Farahbakhsh N Shastri S Sharma P. Biomarkers for diagnosis of neonatal sepsis: a literature review. *J Maternal Fetal Neonatal Med.* (2017) 31:1646–59. 10.1080/14767058.2017.1322060 28427289

[7] Meisner M. *Procalcitonin - Biochemistry and Clinical Diagnosis.* Bremen: UNI-MED Verlag AG (2012).

[8] Chiesa C Panero A Rossi N Stegagno M De Giusti M Osborn JF Reliability of procalcitonin concentrations for the diagnosis of sepsis in critically ill neonates. *Clin Infect Dis.* (1998) 26:664–72. 10.1086/514576 9524841

[9] Altunhan H Annagür A Örs R Mehmetoğlu I. Procalcitonin measurement at 24 hours of age may be helpful in the prompt diagnosis of early-onset neonatal sepsis. *Int J Infect Dis.* (2011) 15:e854–8. 10.1016/j.ijid.2011.09.007 22019570

[10] Chiesa C Natale F Pascone R Osborn JF Pacifico L Bonci E C reactive protein and procalcitonin: reference intervals for preterm and term newborns during the early neonatal period. *Clin Chim Acta.* (2011) 412:1053–9. 10.1016/j.cca.2011.02.020 21338596

[11] Aydemir C Aydemir H Kokturk F Kulah C Mungan AG. The cut-off levels of procalcitonin and C-reactive protein and the kinetics of mean platelet volume in preterm neonates with sepsis. *BMC Pediatr.* (2018) 18:253. 10.1186/s12887-018-1236-2 30068303 PMC6090766

[12] Fukuzumi N Osawa K Sato I Iwatani S Ishino R Hayashi N Age-specific percentile-based reference curve of serum procalcitonin concentrations in Japanese preterm infants. *Sci Rep.* (2016) 6:23871. 10.1038/srep23871 27033746 PMC4817150

[13] Naramura T Tanaka K Inoue T Imamura H Yoshimatsu H Mitsubuchi H New reference ranges of procalcitonin excluding respiratory failure in neonates. *Pediatr Int.* (2020) 62:1151–7. 10.1111/ped.14282 32365428

