# Supplementary material for: Procalcitonin levels in preterm newborns: Reference ranges during the first three days of life
Source: Front Pediatr. 2022 Aug 29;10:925788. doi: 10.3389/fped.2022.925788 (PMC9464813; doi:10.3389/fped.2022.925788)
Supplement: Supplementary file 1 [file Table_1.DOCX]

STUDY DESIGN
